# Sky Compass Orientation in Desert Locusts—Evidence from Field and Laboratory Studies

**DOI:** 10.3389/fnbeh.2015.00346

**Published:** 2015-12-16

**Authors:** Uwe Homberg

**Affiliations:** Faculty of Biology, Animal Physiology, Philipps UniversityMarburg, Germany

**Keywords:** sky compass orientation, animal migration, polarization vision, insect brain, desert locust

## Abstract

Locusts are long-range migratory insects. At high population density, immature animals form marching hopper bands while adults take off and form huge swarms of millions of animals. At low population densities animals are solitarious, but likewise migrate, mostly during the night. Numerous studies aimed at predicting locust infestations showed that migrations both as hopper bands and as adults are largely downwind following seasonal shifts of the tropical convergence zone taking the animals to areas of rainfall. Only a few studies provided evidence for active orientation mechanisms, including the involvement of a sun compass. This scarcity of evidence stands in contrast to recent neurobiological data showing sophisticated neuronal adaptations suited for sky compass navigation. These include a special dorsal eye region with photoreceptors suited to analyze the polarization pattern of the sky and a system of topographically arranged sky compass neurons in the central complex of the brain. Laboratory experiments, moreover, demonstrated polarotaxis in tethered flying animals. The discrepancy of these findings call for more rigorous field studies on active orientation mechanisms in locusts. It remains to be shown how locusts use their internal sky compass during mass migrations and what role it plays to guide solitarious locusts in their natural habitat.

## Introduction

**Figure 1:**
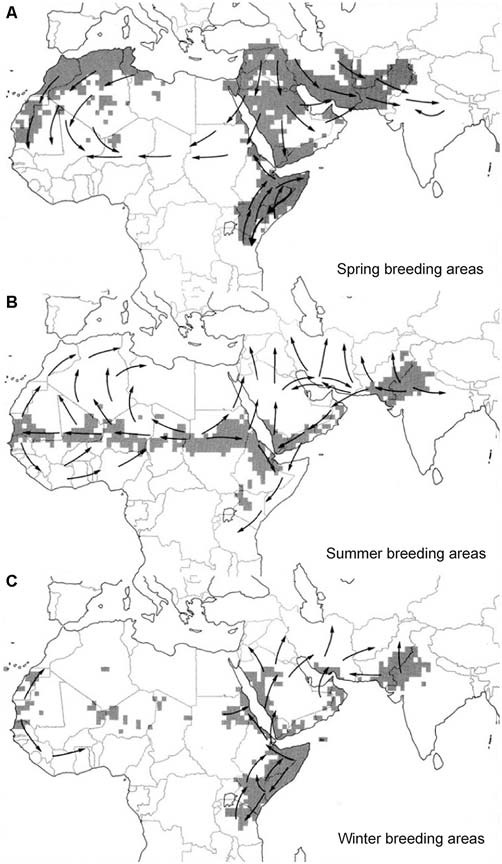
**(A–C)** Seasonal breeding areas (shaded gray) and major movements of desert locust (*Schistocerca gregaria*) swarms (arrows). Adapted from Roffey and Magor (2003) with kind permission.

Many insect species of various orders can become migratory during certain seasons, certain periods of their lives, or under particular environmental conditions (Dingle, 2014; Chapman et al., 2015). One of the most spectacular of these displacements is the mass migration of locusts. Locusts comprise about a dozen species of grasshoppers (*Acrididae*) which can change their behavior and appearance depending on population density (phase polyphenism; Pener and Simpson, 2009). Animals can occur in two phases, a solitarious and a gregarious phase. One of the best studied locust species is the desert locust (*Schistocerca gregaria*) occurring in Africa and parts of Asia (Figure 1). At low population densities, desert locusts are solitarious: they are of cryptic coloration and actively avoid each other (Roessing et al., 1993; Simpson et al., 1999). At higher population densities, animals undergo a number of changes in morphology, physiology, and behavior, the most obvious of which are changes in coloration and aggregation in groups. As immature larva they form marching hopper bands while the winged adults form large migratory swarms. Desert locusts inhabit arid to semi-arid environments with intermittent rainfall changing with long periods of drought. Phase change and migratory behavior are, therefore, regarded as an adaptation to dramatically changing environmental conditions and an effort to colonize new breeding habitats following changing areas of rainfall (Dingle, 1972). Eggs are laid in pods of around 80–150 eggs in moist sand or soil at a depth of about 10–15 cm. Egg development is highly dependent on the presence of free soil water which is absorbed during embryonic development. Therefore, successful breeding implies close association between egg laying and rainfall. As a further adaptation to seasonal changes in rainfall, sexual maturation can be delayed for as much as 6 months until seasonal rains have been encountered.

The migratory behavior of the desert locust has been studied intensely over more than a century in an effort to forecast locust infestations. Desert locusts are the record holder in swarm size (up to several billion animals in a single swarm) and travelling distance (up to 5000 km) and have caused most devastating damage to crops across North Africa (Uvarov, 1977).

## Migration of Gregarious Locusts

Migration is a prominent feature of desert locust biology. Depending on the seasonal occurrence of rainfall locust populations move between spring, summer and winter breeding areas (Figure 1). Under favorable conditions, population densities increase and at densities of 10–15 individuals per m^2^, animals begin to aggregate in marching bands as nymphs and airborne streams and swarms as adults (Uvarov, 1977). This goes along with a switch in activity from nocturnal (solitarious) to diurnal (gregarious). Locust streams fly at low altitude in diffuse formation, whereas larger swarms advance over a broad front. Gregarious animals spend the night roosting in vegetation and take off spontaneously in the morning. The progression of swarms occurs in a characteristic form of rolling motion (Gunn et al., 1948; Uvarov, 1977). Animals at the forefront of the swarm settle down for feeding and resting and resume flight near the end of the swarm, when the number of overflying animals declines. Flight speed of individual animals is around 3–6 m/s, and whole swarms may cover a daily distance of 5–130 km depending on meteorological conditions, like temperature and wind speed (Rainey, 1963; Uvarov, 1977). The direction of individual fliers may be highly variable, but neighbors largely fly in parallel, and locusts at the edge tend to fly into the swarm, apparently attracted by aggregation pheromones (Obeng-Ofori et al., 1994; Torto et al., 1994). Visual and accoustic signals may, likewise, contribute to swarm cohesion (Uvarov, 1977; Spork and Preiss, 1994).

**Figure 2:**
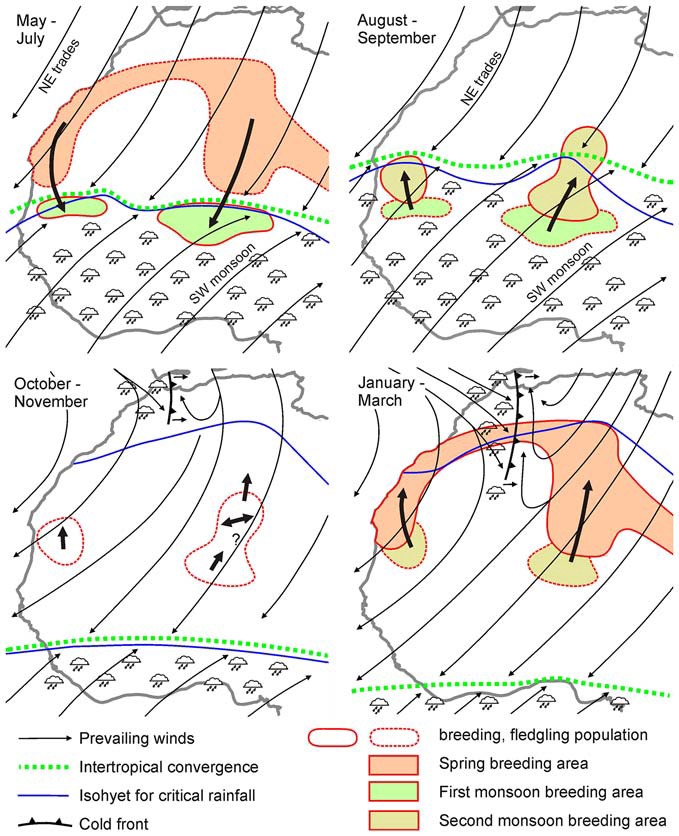
**Seasonal movements of different generations of desert locust populations in West Africa in relation to seasonal shifts in the Intertropical Convergence Zone (ITCZ), monsoons and winter rains.** Note that migratory directions from January—March (lower right) are against the prevailing wind directions. Data are largely based on migrations of individuals rather than swarms. Adapted from Farrow (1990) after Popov (1965) with kind permission.

Swarm movements follow a more or less regular annual cycle with back and forth movements in many areas and circling routes in others (Figure 1). Swarming locusts often fly downwind (Gunn et al., 1948), but up-wind or cross-wind movements have also been observed, especially if wind speed was lower than locust air speed (Kennedy, 1951; Baker et al., 1984). In the latter situation, swarms usually flew at low altitude, avoiding stronger winds in the upper atmosphere or even settled on the ground during strong headwind (Kennedy, 1951). In a pioneering study to forecast locust outbreaks, Rainey (1951, 1963) analyzed swarm movements in eastern Africa and showed that these migrations largely correspond with seasonal shifts of the Intertropical Convergence Zone (ITCZ; Figure 2). The ITCZ is a zone of converging trade winds and monsoon currents originating on opposite sides of the equator with high probability of rainfall (Figure 2). It lies near the equator in January and reaches its most northerly position near the tropic of cancer in August (Figure 2), largely following the seasonal shift in the sun’s zenith position. Swarming behavior of desert locusts following downwind directions is, therefore, most likely an adaptation of desert locusts to be carried to areas of rainfall and thus suitable breeding grounds (Dingle, 2014). Deviations of swarm track directions from the prevailing downwind directions led Baker (1978) to propose that swarms rather orient in particular compass directions than relative to the wind. This hypothesis, however, was subsequently dismissed by Draper (1980), who found no evidence for preferred compass directions based on re-analysis of the evaluated flight tracks of Baker.

## Activity of Solitarious Locusts

During drought periods, termed recessions, desert locusts occur in patchy distribution in arid areas of the Sahel zone in northern Africa, across the Arabian Peninsula and as far east as northwest India (Uvarov, 1977). Under these conditions, animals occur at low population densities (<10^3^ per km^2^) and as laboratory experiments showed, actively avoid each other (Roessing et al., 1993; Simpson et al., 1999). In this so called solitarious phase, the animals are largely nocturnal, their coloration is uniformly green in the larval stage, and cryptically pale green to yellowish as adults. Little is known about movements and activity patterns of immature larva, termed hoppers. Adult animals rest on the ground or roost in vegetation during the day and only fly when they are disturbed. Animals are more active after dusk and may take off for short distance or migratory flight 20–30 min after sunset. Flight activity peaks 1–2 h after sunset and then declines gradually (Roffey, 1963; Waloff, 1963; Schaefer, 1976; Farrow, 1990). Flights may be of short distance (less than 1 km) and associated with local movements, others are more sustained and can cover distances of 60–250 km in a single night. Migration and oogenesis are apparently closely associated (Farrow, 1990). In poor, dry conditions, movements are generally restricted to local dispersals, but flight activity increases substantially at the onset of drought breaking rain (Farrow, 1990). Substantial emigration may, likewise, not occur if conditions in fledging areas are locally suitable for further reproduction, leading to an increase in population size and finally, transition to the gregarious phase (Roffey and Popov, 1968).

At low wind speeds (<2 m/s) flight tracks, observed visually, are apparently independent of wind directions, while at wind speeds above 2–4 m/s tracks are predominantly downwind (Roffey, 1963; Waloff, 1963). A remarkable feature of night-flying locusts and other acridoids is that animals often exhibit a considerable degree of mutual alignment and common orientation (Schaefer, 1976; Riley and Reynolds, 1979, 1983, 1986). Radar observations of a variety of species showed that the orientation directions of migrating individuals were partly related to wind directions. Whereas Schaefer (1976) reported that orientations of desert locusts in the Sahara were almost always downwind, Riley and Reynolds (1986) observed that even at wind speeds above the insects’ flight speed (about 3 m/s), the degree of collective flight directions differed as much as 90° from the wind direction leading to significant wind drifts. Common compass bearings to preferred geographic directions occurred at low wind speeds but the sensory basis remained unclear. Orientations were maintained even when the sun and moon were well below the horizon and even on moonless nights (Riley and Reynolds, 1986). Therefore, direct moonlight or the night sky polarization pattern caused by the moon were ruled out as orientation cues for that behavior. Recent evidence from dung beetles navigating with respect to the milky way or night sky polarization pattern (Dacke et al., 2013; el Jundi et al., 2015) may, however, justify to reevaluate the role of visual signals as orientation cues during nighttime flights.

The complexity of migrations in relation to meteorological data is illustrated in Figure 2 for solitarious populations in West Africa. Downwind displacements of successive generations in May to September largely follow the seasonal shifts in the ITCZ, a major belt area of rainfall (Figure 2). In October–November, however, animals do not follow the ITCZ further south but disperse locally, and from January–March move polewards against the direction of prevailing winds (Figure 2), suggesting some form of active orientation.

## Sun Compass Orientation

Many observers agree that active orientation underlies at least in part desert locust migrations (Kennedy, 1951; Baker et al., 1984; Riley and Reynolds, 1986; Farrow, 1990) although a general theory of swarm behavior is not at hand. Although the general view is that swarms move downwind, frequent observations of flight courses deviating significantly from prevailing wind directions as well as the stability of orientation when deflected by temporary wind gusts argue for active control of the animals on their orientation. Active orientation has also been concluded from the relatively regular seasonal shifts of swarms and solitarious locusts occurring as circular or back and forth movements in different parts of Africa and the Middle East (Figures 1, 2).

Experimental evidence for a sun compass orientation as part of the migration strategy of the locust was provided by Kennedy (1945, 1951). Marching hopper bands move in rather constant direction from dawn to dusk and usually maintain walking direction over several days, especially in unobstructed terrain (Kennedy, 1945; Ellis and Ashall, 1957). Different bands in the same area may move in parallel or in different directions, but Kennedy found no evidence for a particular orientation relative to wind directions. Within a band there is a strong tendency for individual animals to follow the common marching direction, which Kennedy termed “gregarious inertia”. To study the role of the sun as a guiding factor, he obscured the sun with a blanket and artificially altered the position of the sun in the shaded area by a mirror. Most dramatic effects were observed when the sun’s reflected image was shifted by 180° (e.g., from the right to the left). Animals stopped, turned around and marched in the opposite direction. Animals could be made to walk alternately in opposite directions by showing or removing the blanket and mirror. Because of gregarious inertia, the experiments were performed on thin streams of marching animals and not at a point of densely crowded hoppers, where the tendency to follow the direction of animals not affected by the stimulus would be in conflict with the individual’s sun compass orientation. The data strongly suggest that the tendency to walk in a common direction (gregarious inertia) together with a sun compass mechanism determines the stability of marching direction in areas without obstacles. An internal time compensation mechanism apparently compensates for daytime shifts in solar position, as has been found in many animals using sky-compass orientation (review: Guilford and Taylor, 2014; honeybees: Lindauer, 1960; desert ants: Wehner and Lanfranconi, 1981; butterflies: Perez et al., 1997; Oliveira et al., 1998). In contrast the choice of marching directions appears to be determined by other factors such as particular features of the terrain or common orientation to the morning sun (basking) before the start of band formation (Kennedy, 1945). Ellis and Ashall (1957) repeated Kennedy’s mirror experiments, however with mixed results. Only few animals turned around, and the authors interpreted this as a thermal reaction to reflected heat from the mirror rather than a sun compass response.

Kennedy (1951) performed similar experiments on flying individuals in a swarm: changing the sun’s image from the right to the left side by a large mirror as locusts flew through the shadow of a tree led to a turnaround of the animals continuing flight in opposite direction as long as they were in the tree’s shadow and exposed to the reflected sun. As a note of caution it should be mentioned that these experiments could be performed on only five animals. In addition, migrants when thrown off from their course by disturbances reverted to their original course soon after, even if isolated from other migrants. Taken together, these experiments by Kennedy provide clear evidence for a sun compass orientation in larval and—with some reservation—in adult migrants, although no quantitative data were collected. Surprisingly, except for attempts by Ellis and Ashall (1957), Kennedy’s experiments have never been repeated or extended. Accordingly, the effects of sun-derived celestial cues on migratory directions such as the polarization pattern of the sky or the sky chromatic contrast have never been tested in the field.

## Laboratory Studies—Behavior

In contrast to rather weak direct evidence for sun compass orientation of locusts in the field, behavioral experiments in the laboratory as well as neurobiological studies provide strong support for the ability of locusts to navigate using a sky compass. Especially the ability to detect the plane of dorsally presented polarized light strongly suggests that locusts, like many other insects species, can detect the sky polarization pattern and use it—together with other sun-derived celestial signals—as a compass for spatial orientation.

**Figure 3:**
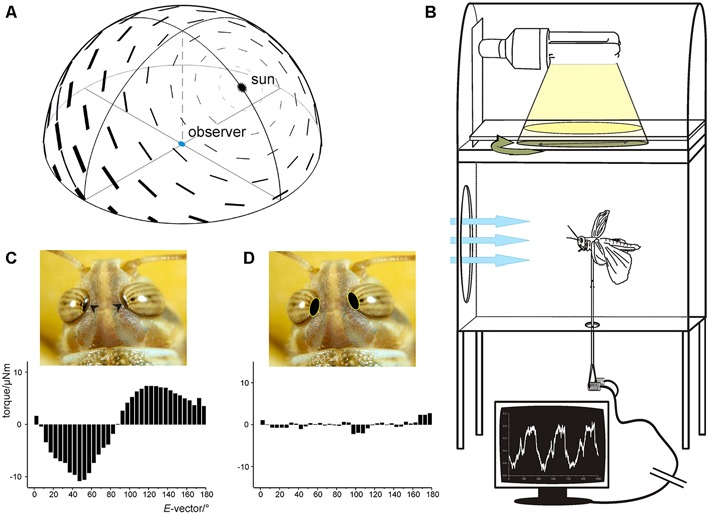
**(A)** The celestial polarization pattern at a solar elevation of 50°. *E*-vectors of plane polarized light are arranged tangentially to concentric circles around the sun. The degree of polarization (bar thickness) is maximal at 90° from the sun. **(B,D)** Polarotaxis of the desert locust. **(B)** Experimental setup. The animal is mounted on a vertical rod attached to a yaw-torque meter. Tethered flight is initiated by laminar frontal wind (blue arrows). Yaw torque is measured while the animal is stimulated with white light from above passing through a rotating polarizer. **(C)** Averaged data of four 360° rotations of the polarizer reveals periodic changes in yaw torque corresponding with the periodicity of the polarizer. **(D)** When the dorsal rim areas of both eyes (arrowheads in **C**) are covered with black paint, yaw torque becomes irregular and no longer corresponds to the position of the polarizer. **(A)** from Pfeiffer and Homberg (2014) with kind permission; **(B)** from Backasch (2009) with kind permission; **(C,D)** from Homberg (2004).

Two assays have demonstrated polarotactic behavior when illuminating locusts from dorsal directions. Eggers and Weber (1993) showed that locust larva walking on a Kramer sphere orient themselves menotactically with respect to polarized light presented from dorsal direction, i.e., they maintained constant but individually different body orientation relative to the orientation of the polarizer. Likewise, tethered flying adults showed periodic yaw-torque responses when illuminated from dorsal direction through a slowly rotating polarizer, corresponding to the 180° periodicity of the stimulus (Figure 3; Mappes and Homberg, 2004). Those experiments were performed on laboratory-raised animals without navigational experience. This might be the reason why only a fraction of animals showed consistent responses to the rotating polarizer (Mappes and Homberg, 2004). Like in walking larva, there was no common preferred orientation relative to the orientation of the polarizer among 82 animals tested. Interestingly, the polarotactic response was abolished after covering distinct dorsal rim areas of the eyes with black paint. This result shows that locusts, like many other insect species (Labhart and Meyer, 1999) detect zenithal polarized light through a specialized dorsal rim area, which is particularly prominent in *S. gregaria* and unlike in other species is visible to the naked eye (Figure 3).

## Laboratory Studies—Neurobiology

The neural mechanisms in the locust brain involved in the processing of polarized light signals have been analyzed in detail and provide further evidence for the ability of these insects to use sun-related cues in the sky as compass signals for spatial orientation (Homberg et al., 2011). The dorsal rim area is highly specialized for polarization plane detection. All photoreceptors are blue sensitive (Schmeling et al., 2014). Thereby, differences in signaling amplitude between different photoreceptors cannot be based on differences in spectral sensitivity of the receptors. In contrast to the main retina, the microvilli of a given photoreceptor in the dorsal rim area are highly aligned (Homberg and Paech, 2002) resulting in high sensitivity to the plane of light-wave oscillation (*E*-vector). The ratio of absorption of light oscillating parallel to the microvillar axis to absorption perpendicular to it (PS-value) is low in the main eye (1–4) but up to over 30 in the dorsal rim area (Schmeling et al., 2014) owing to high alignment of the individual microvilli of the rhabdomere. Photoreceptors in each ommatidium form two sets of microvillar orientations orthogonal to each other; therefore each dorsal rim ommatidium can be regarded as a system of cross-analyzers for the plane of polarization above the animal. The receptive fields of dorsal rim photoreceptors are particularly large (acceptance angle about 33°) and show considerable overlap between adjacent ommatidia. When combining the receptive fields of individual photoreceptors measured across the dorsal rim area, the resulting visual field of both dorsal rim areas covers almost the entire sky (Schmeling et al., 2015).

**Figure 4:**
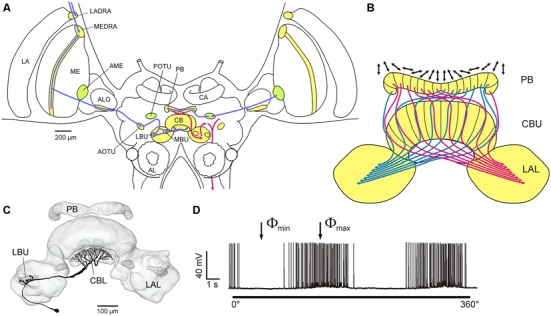
**Central processing of polarized light signals from zenithal directions in the locust brain. (A)** Polarization vision pathways in the brain of the desert locust. Processing stages for polarization analysis include dorsal rim areas in the lamina and medulla (LADRA, MEDRA), a ventral layer in the anterior lobe of the lobula (ALO), the anterior optic tubercle (AOTU), the lateral and medial bulbs of the lateral complex (LBU, MBU), the central body (CB) and the protocerebral bridge (PB). In a second pathway, the accessory medulla (AME) is connected to the PB via the posterior optic tubercle (POTU). Inputs to the central complex are shown in blue, outputs in red. AL, antennal lobe; CA, calyx of the mushroom body. **(B)** Idealized compass-like representation of *E*-vector tunings (double arrows) in columnar neurons of the PB and upper division of the central body (CBU). LAL, lateral accessory lobe. **(C,D)** Morphology **(C)** and polarized light sensitivity **(D)** of a tangential neuron of the lower division of the central body (CBL). When the animal is illuminated from above through a rotating polarizer, spike frequency is modulated as a function of *E*-vector orientation. Maximum (Φ_max_) and minimum spiking activity (Φ_min_) occurs at orthogonal *E*-vectors. **(A,B)** adapted from Pfeiffer and Homberg (2014); **(C,D)** from Heinze et al. (2009) with kind permission.

Signals from the dorsal rim area of the eye are processed via several stages in the optic lobe and central brain and finally converge from both eyes in the central complex, a midline-spanning neuropil consisting of several substructures (Figure 4A). Many neurons along this pathway and in the central complex show polarization opponency (Figure 4D), i.e., the neuron is maximally excited by light polarized in a particular plane (Φ_max_) and is maximally inhibited by light polarized in the orthogonal plane (Φ_min_). Therefore, these neurons receive antagonistic input from two *E*-vector analyzers with orthogonal sensitivity, likely to be represented by the two sets of photoreceptors with orthogonal microvilli orientations in the dorsal rim area. In the central complex an extensive network of neurons is involved in polarized light processing and generates a compass-like topographic representation of neuronal *E*-vector-tunings. The protocerebral bridge (PB) and the upper and lower divisions of the central body, the three major components of the central complex, are organized into series of 16 slices from right to left. Systems of columnar neurons connect individual slices of different subdivisions and send axonal projections to the lateral accessory lobes, the major output targets of the central complex (Figure 4B). Comparing the *E*-vector tuning to zenithal polarized light of individual columnar neurons innervating different slices revealed a compass-like arrangement of Φ_max_ orientations covering 2 × 180° thoughout the 16 slices (Heinze and Homberg, 2007). If this system is indeed used to analyze the polarization pattern of the sky it could inform the animal about its current orientation relative to the solar azimuth (Homberg et al., 2011), and might therefore, be the insect equivalent of head direction cells in the hippocampal formation of rats (Taube, 2007). Probing the responses to polarized light stimuli from 37 positions in the dorsal hemisphere showed that central complex neurons have receptive fields covering the whole sky (Bech et al., 2014). Moreover, *E*-vector tuning varied position dependently in a systematic way closely matching the polarization pattern of the sky for certain solar positions (Bech et al., 2014). These data, in addition, show that full analysis of the sky polarization pattern instead of a single *E*-vector orientation in the zenith may provide the locust with unbiased information on solar azimuth. Projection neurons from the lateral accessory lobes, finally, directly or indirectly contact neurons descending from the brain to thoracic ganglia (Figure 4). These neurons are strong candidates for direct control of flight motor output (Heinze and Homberg, 2009; Träger and Homberg, 2011). The key role of the central complex in sky polarization analysis is not unique to locusts but is also supported by studies in the field cricket (Sakura et al., 2008), the monarch butterfly (Heinze and Reppert, 2011), and two species of dung beetles (el Jundi et al., 2015).

In addition to the pattern of polarization, the position of the sun as the brightest spot in the sky and the chromatic gradient can be used a compass signals, as demonstrated in several insect species. Polarized-light sensitive neurons of the locust polarization vision pathway, beginning in the medulla of the optic lobe, indeed, receive additional unpolarized visual input, apparently through photoreceptors of the main eye suggesting an integration of all sky signals for a robust coding of solar azimuth (Pfeiffer and Homberg, 2007; el Jundi et al., 2014).

Maintaining the same direction of migration over several days as has been observed in the field requires a time-compensating mechanism if a sky compass is relied on for navigational directions. Basically, inputs from an internal circadian clock have to modulate the output of an internal compass to compensate for the shift of the solar azimuth during the day. The site of the circadian clock in the locust brain is unknown but comparative analysis in flies and cockroaches have identified a neural network in the accessory medulla (AME) of the brain as the site of the circadian pacemaker controling locomotor behavior (Helfrich-Förster et al., 1998). Interestingly a pathway originating in the AME of the desert locust provides input to the protocerebral bridge (Figure 4A), but the nature of these signals has still to be uncovered.

Solitarious locusts migrate at night, but a marked increase in absolute sensitivity of photoreceptors (Schmeling et al., 2014, 2015) or interneurons of the polarization vision pathway (el Jundi and Homberg, 2012) has not been found in solitarious animals. Whether their visual system is sensitive enough to detect the polarization pattern generated by the moon, as has been demonstrated recently for nocturnal dung beetles (el Jundi et al., 2015), or even the milky way for orientation purposes, again demonstrated for nocturnal dung beetles (Dacke et al., 2013), will have to await future studies.

## Synopsis

Work demonstrating an elaborate neural basis for sky compass orientation in desert locusts considerably strengthens evidence from field experiments for the existence and use of a sun compass involved in maintaining walking and flight direction in desert locusts. At minimum, the sun compass might have a stabilizing effect on the maintenance of migratory directions allowing the animals: (1) to resume their original direction after being diverted by obstacles, wind gusts etc., and (2) allow for maintenance of migratory directions of several days. Its use and interaction with other factors such as wind direction, has not been studied properly, but might allow the animals to fly against prevailing wind directions as has been reported for swarms and solitarious animals. As Uvarov pointed out “there is no evidence of any inherent urge to migrate in any particular direction…” (Uvarov, 1977, p. 346), so once a direction has been chosen, a time compensated sky compass would be essential to maintain it for maximum efficiency in distance coverage. Given the wealth of laboratory evidence, it will be important in the future to more clearly investigate the role of different celestial cues on navigation direction in both solitarious and gregarious larva and adults in the field.

## Author Contributions

Text and figures were compiled by the author.

## Funding

Supported by DFG grants HO 950/16, HO 950/21 and HO 950/23.

## Conflict of Interest Statement

The author declares that the research was conducted in the absence of any commercial or financial relationships that could be construed as a potential conflict of interest.
